# The antioxidant response of the liver of male Swiss mice raised on a AIN 93 or commercial diet

**DOI:** 10.1186/1472-6793-13-3

**Published:** 2013-01-24

**Authors:** Aline C Caetano, Lucimara F da Veiga, Flávia R Capaldi, Severino M de Alencar, Ricardo A Azevedo, Rosangela MN Bezerra

**Affiliations:** 1 Postgraduate Program in Food Science and Technology, Escola Superior de Agricultura “Luiz de Queiroz”, University of São Paulo (USP), Piracicaba, SP, Brazil; 2 Department of Agri- Food Industry, Food and Nutrition, “Luiz de Queiroz” College of Agriculture, University of São Paulo (USP), Piracicaba, SP, Brazil; 3 Department of Genetics, “Luiz de Queiroz” College of Agriculture, University of São Paulo (USP), Piracicaba, SP, Brazil; 4 School of Applied Sciences, University of Campinas (UNICAMP), 1300, Pedro Zaccaria St, Jd Sta Luiza, 13484-350, Limeira, São Paulo, Brazil

**Keywords:** AIN 93 diet, Antioxidant enzymes, Commercial diet, Lipid peroxidation, Liver, Mice, Oxidative stress, Reactive oxygen species

## Abstract

**Background:**

Reactive oxygen species (ROS) are formed under natural physiological conditions and are thought to play an important role in many human diseases. A wide range of antioxidants are involved in cellular defense mechanisms against ROS, which can be generated in excess during stressful conditions, these include enzymes and non-enzymatic antioxidants. The aim of this study was to evaluate the antioxidant responses of mice to two diets control, commercial and the purified AIN 93 diet, commonly used in experiments with rodents.

**Results:**

Malondialdehyde (MDA) and hydrogen peroxide (H_2_O_2_) concentrations and superoxide dismutase (SOD) and glutathione reductase (GR) activities determined in the liver were lower in the group of mice fed with the AIN 93 diet, while catalase (CAT) activity was higher in the same group, when compared to the group fed on the commercial diet. Liver glutathione peroxidase (GSH-Px) activity was similar in the groups fed on either AIN 93 or the commercial diets. Two SOD isoforms, Mn-SODII and a Cu/Zn-SODV, were specifically reduced in the liver of the AIN 93 diet fed animals.

**Conclusions:**

The clear differences in antioxidant responses observed in the livers of mice fed on the two diets suggest that the macro- and micro-nutrient components with antioxidant properties, including vitamin E, can promote changes in the activity of enzymes involved in the removal of the ROS generated by cell metabolism.

## Background

Differences in the composition of antioxidant compounds in diets and nutritional supplements are known to cause changes in the responses of enzymes involved in cellular defense mechanisms against free radicals [1,2]. Experimental studies using a commercial diet for rodents as a control diet, revealed a smaller antioxidant response in the control animals when compared to obese animals grown on a high fat diet. It was suggested that such a difference may be due to the amount of vitamins, such as vitamin E in the diet [3]. Under normal metabolic conditions, components of the defense mechanism of the liver, such as the enzymes superoxide dismutase, catalase and glutathione peroxidase, and non-enzymatic antioxidants such as glutathione, vitamin A, C and E, may prevent the accumulation of intracellular free radicals [4] and eventual cellular damage. The presence of vitamins and other nutrients with antioxidant activity, acting in conjunction with antioxidant enzymes has been shown to have beneficial effects against free radicals produced under normal physiological and pathophysiological conditions [5].

Although an aerobic existence provides many advantages, the use of oxygen by cells results in the production of free radicals, which can be defined as molecules or molecular fragments containing one or more unpaired electron in atomic or molecular orbitals [6]. Reactive oxygen species (ROS) are defined as oxygen-containing molecules, which may or may not have unpaired electrons, but are highly reactive in biological tissues. In recent years it has become apparent that low concentrations of hydrogen peroxide (H_2_O_2_) may be required for normal cellular function and intracellular signaling. Physiological ROS are generated at the plasma membrane and endomembranes by NADPH oxidase [7].

Studies have shown that the presence of antioxidants in the diet increases the cellular defense mechanisms, reducing the levels of ROS generated during cell metabolism to normal cell conditions [8]. In this study, the effect of a purified AIN 93 diet and a commercial diet on the antioxidant responses of the liver of male Swiss strain mice, were compared.

## Methods

### Animals and diets

Three-week-old male Swiss strain mice (Sw/Uni) free of specific pathogens were obtained from the State University of Campinas Breeding Center (CEMIB-UNICAMP, Brazil); the animals were housed in individual cages at 20°C with a 12 h light/ 12h dark cycle and were given free access to the diet and water. Six mice were fed with a commercial diet for rodents (CD) and six were fed with the AIN 93 purified diet for 56 days. The commercial diet contained 25.6% kcal protein, 62.6% kcal carbohydrate, 11.8% kcal lipid and 0.006% diet vitamin E (NUVILAB- CR1, Nuvital Nutrientes S/A), whereas the AIN 93 purified diet contained 19.9% kcal protein, 64.4% kcal carbohydrate, 15.7% kcal lipid and 0.015% vitamin E [9] (Table 1). The animals were anesthetized, and after loss of corneal and paw reflexes, the liver tissue was collected. All mice experiments were approved by the Bioethics Committee of Odontology College of Piracicaba (FOP / UNICAMP), under protocol n° CEEA 888–1.

**Table 1:** Commercial and AIN 93 diets compounds and chemical composition

	**NUVILAB**- **CR1**- **Nuvital**		**AIN 93 G**/**M**	
whole corn ground		casein	
soy bran		dextrinized corn starch	
wheat bran		corn starch	
calcium carbonate		cellulose	
dicalcium phosphate		mineral mix	
sodium chloride		vitamin mix	
vitamin and mineral mix		L-cystine	
amino acids		choline bitartrate	
		soy oil	
		sucrose	
Proteins (min)	25.6% kcal	Protein	19,9% / 14.5% kcal
Lipids (min)	11.8% kcal	Lipids	15.7% / 9.3% kcal
Carbohydrate	62.6% kcal	Carbohydrate	64.4% / 76.2% kcal
Vitamin E	0.006 g%	Vitamin E	0.015 g%

### Lipid peroxidation

Lipid peroxidation was determined by estimating the content of thiobarbituric acid reactive substances (TBARS) following the method of Heath & Packer [10]. The concentration of malondialdehyde (MDA) equivalents was calculated using an extinction coefficient of 1.55 × 10^-5^.mol^-1^.cm^-1^.

### Hydrogen peroxide concentration

H_2_O_2_ was measured spectrophotometrically (Lambda 40, Perkin Elmer) after reaction with potassium iodide (KI) [11]. The reaction mixture consisted of 0.2 mL 0.1% trichloroacetic acid (TCA) containing the liver extract supernatant, 0.2 mL of 100 mM K-phosphate buffer and 0.8 mL reagent (1 M KI (w/v) in fresh double-distilled water). The blank consisted of 1% TCA in the absence of liver extract. The reaction was developed for 1h in darkness at room temperature and the absorbance measured at 390 nm. The amount of H_2_O_2_ was calculated using a standard curve prepared with known concentrations of H_2_O_2_.

### Extraction, determination of protein concentration and analysis of antioxidant enzymes

The following steps were carried out at 4°C unless stated otherwise. The liver tissue was homogenized (5:1 buffer volume: fresh weight liver) in a mortar with a pestle with 100 mM potassium phosphate buffer (pH 7.5) containing 1 mM ethylenediaminetetraacetic acid (EDTA) and 3 mM DL-dithiothreitol [12]. The homogenate was centrifuged at 12,100 × g for 30 min and the supernatant was kept stored in separate aliquots at −80°C, prior to the determination of protein concentration, superoxide dismutase (SOD), catalase (CAT), glutathione peroxidase (GSH-Px) and glutathione reductase (GR) activity. The protein concentration of all the samples was determined by the method of Bradford [13] using bovine serum albumin as a standard. SOD activity was determined as described by Giannopolitis & Reis [14] and the SOD isoform determination was carried out as described by Azevedo et al. [12], following native polyacrylamide gel electrophoresis (PAGE). CAT and GR activities were assayed as described by Cia et al. [15]. GSH-Px was determined as described by Flohé & Günzler [16].

### Statistical analysis

The data are reported as means ± standard error of the mean (SEM). Statistical analysis was performed by an unpaired two-tailed *t* test, Mann Whitney test using GraphPad Prism 6 software. *P* <0.05 was considered statistically significant.

## Results

**Figure 1:**
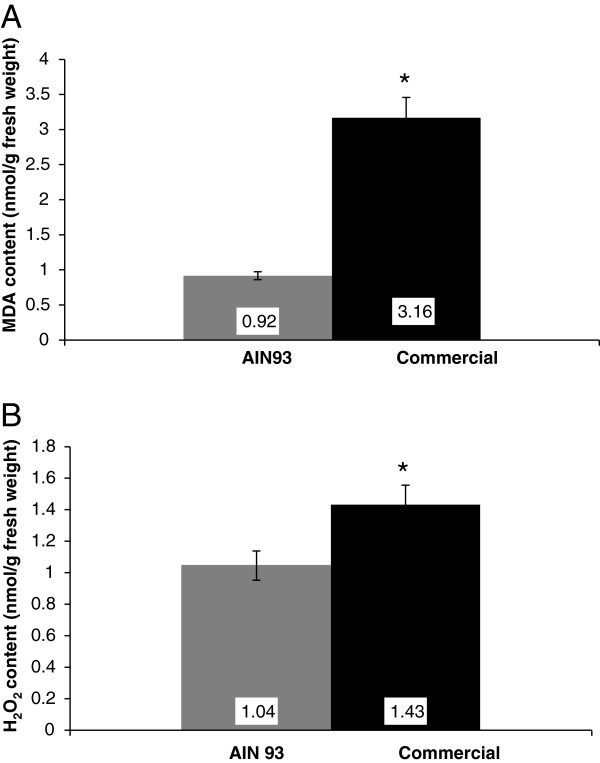
**Lipid peroxidation (MDA content) and hydrogen peroxide concentration in the livers of male Swiss mice fed on two different diets.** The mice were fed with AIN 93 purified diet (AIN 93) or commercial diet for rodents (CT) for 56 days. The animals were anesthetized, and after loss of corneal and paw reflexes, the liver was collected. Lipid peroxidation was determined by estimating the content of thiobarbituric acid reactive substances (TBARS) and H_2_O_2_ was measured spectrophotometrically after reaction with potassium iodide in liver tissue. MDA (**A**) and hydrogen peroxide content (**B**) of the livers of animal fed with AIN 93 and commercial diet for 56 days. *P* < 0.05, unpaired two-tailed *t* test, Mann Whitney test. Each value is expressed as mean ± SEM.

The concentration of MDA was used as a biomarker of lipid peroxidation (Figure 1A). The results revealed that the concentration of MDA was higher in the livers of the commercial diet fed mice (*P* = 0.0022) than in the AIN 93 fed group. The concentration of H_2_O_2_ (Figure 1B) was also higher in the same commercial group (*P* = 0.0152).

**Figure 2:**
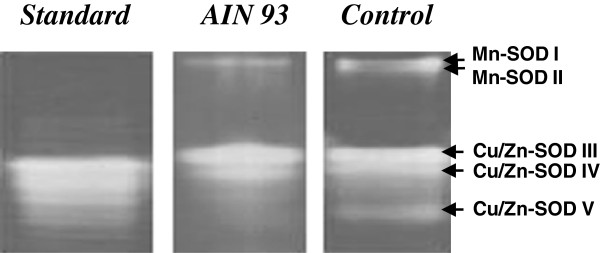
**SOD isoforms of male Swiss mice fed on two different diets.** Electrophoresis was performed as described in Materials and Methods. The gel was placed in a dark box with dye solution for 30 min. After incubation, the gel was illuminated and kept in water until the development of colorless bands of SOD activity in a purple-stained gel was visible. The picture illustrates the **i**dentification of SOD isoforms in the livers of mice fed with a AIN 93 or commercial diet. Five predominant SOD isoforms were identified in the liver of commercial diet fed animals, whereas only SOD isoforms I, III and IV were clearly visible in AIN 93 diet fed animals.

Up to five distinct SOD isoforms were identified following native PAGE of liver extracts of the mice fed on the two different diets (Figure 2). These were characterized as: Mn-SOD (Bands I and II) that were resistant to KCN and H_2_O_2_, a class of SOD isoforms that has been shown to be present in mitochondria and three Cu/Zn-SOD isoforms (III, IV and V), that were inactivated in the presence of KCN and H_2_O_2_, which are normally located in the cytoplasm. The SOD activity band intensity of Mn-SOD II, Cu/Zn-SOD V and to a lesser extent Mn-SOD1 were considerably reduced if not absent, following PAGE of the liver extracts of AIN 93, when compared to the commercial diet fed animals. Cu/Zn-SOD III and IV were present in the livers of mice fed on both diets and did not exhibit any apparent differences in band intensity following PAGE. The reduced band intensity of the three SOD isoforms detected following PAGE, could readily explains the lower total SOD activity detected in the liver extracts of the AIN 93 fed mice, when compared to those fed on the commercial diet, as shown in Table 2.

**Table 2:** **Superoxide dismutase** (**SOD**), **catalase** (**CAT**), **glutathione**-**peroxidase** (**GSH**-**Px**) **and glutathione reductase** (**GR**) **enzyme activities in the livers of male Swiss mice fed on two different diets**

**Diet**	**SOD(units/mg protein)**	**CAT(μmol/min/mg protein)**	**GSH**-**Px(μmol/min/mg protein)**	**GR(μmol/min/mg protein)**
AIN 93	654.9 ± 5.3	203.1 ± 5.8	7.2 ± 0.3	0.4 ± 0.0
Commercial	106.1 ± 0.7	154.9 ± 3.1	6.3 ± 0.5	0.7 ± 0.0
*P values*	0.0022	0.0022	0.3052	0.0022

Results are expressed as mean ± standard error of the mean (SEM). n = 6 for both diets.

To assess the next stage of the antioxidant defense system, which is the degradation of H_2_O_2_ produced by the SOD reaction, or any other metabolic reactions that may produce excess H_2_O_2_, the activities of three antioxidant enzymes in the livers of the mice subjected to the two diets were determined and are shown in Table 1. CAT activity was higher in the livers of the AIN 93 fed animals than in the commercial diet fed animals. GSH-Px, in a similar manner to CAT, exhibited an increased activity trend in the livers of AIN-93 fed animals when compared to the commercial fed animals, but such an increase was not statistically significant. In contrast, GR activity was lower in the livers of the AIN 93 fed group when compared to the commercial diet group.

## Discussion

A large number of studies have shown that ROS can act as intracellular signaling molecules in many normal physiological processes; however, an increase in such ROS can lead to an imbalance causing oxidative stress [6]. Several factors such as the diet composition, increased energy intake, intense physical exercise, and hypercatabolic conditions are associated with increased cellular levels of ROS and /or decrease in antioxidant defense systems triggering a state of oxidative stress in different tissues/organs [17,18]. The oxidative process such as lipid peroxidation of biomembranes, produces several compounds that are used as molecular markers, among them MDA is one of the most widely used indicators of the cellular redox state [19]. In this study, the concentration of the lipid peroxidation marker MDA in the liver was higher in the group fed with the commercial diet. According to Sohet et al. [3], commercial diets contained lower levels of antioxidant vitamins such as vitamin E, resulting in higher values of MDA in hepatic tissue of animals. In another report using mice treated with 0.004, 0.008 and 0.032% vitamin E, a progressive decrease in MDA levels was detected [20]. In a study where male Wistar rats were submitted to exhaustive stress and treated with gavage administration of vitamin E, decreased production of MDA in the kidney tissue was observed when compared with the control group [2].

As well as MDA, the concentration of H_2_O_2_ has also been used as an indicator of oxidative stress. High concentrations of H_2_O_2_ are closely related with lipid peroxidation, where the H_2_O_2_ in the cell can be converted by the Fenton reaction to the hydroxyl radical, a highly reactive compound involved in the initiation of lipid peroxidation [21]. H_2_O_2_ can be formed from the degradation of superoxide produced during aerobic respiration, and by the exposure of cells to physical, chemical and biological agents [7].

Venditti et al. [22] showed that vitamin E decreased H_2_O_2_ release during basal respiration. This effect led to reduced ROS flow from the mitochondria to the cytosol, limiting oxidative damage to the liver. In this current study, the vitamin E content of the AIN 93 diet was 2.5-fold higher (0.015%) than that of the commercial diet (0.006%), which might suggest participation of Vitamin E, along with other nutrients in the diet, in the responses observed. For instance, in the livers of the animals fed with AIN 93 there was less accumulation of H_2_O_2_, when compared to the animals on the commercial diet, indicating that vitamin E may be interfering in ROS levels during normal cell metabolism.

Vitamin E is an important fat-soluble antioxidant in the body and operates with some of the antioxidant enzymes tested in this study, such as superoxide dismutase (SOD), catalase (CAT) and glutathione peroxidase (GSH-Px), to protect cells from attack by ROS [23]. SOD provides the first line of defense against oxygen derived free radicals [6]. Under stress conditions, high SOD activity reflects a compensatory mechanism to reduce the superoxide radical. Male rats Wistar fed with the control diet supplemented with 0.01% of vitamin E showed a reduction in SOD activity [24]. In the results presented here, there was lower SOD activity in the livers of the AIN 93 fed group compared to the commercial diet group, suggesting that vitamin E might play an important role in lipid peroxidation and, indirectly, in regulating SOD activity by maintaining a reduced level of superoxide in the cell system. The SOD isoforms II and V were hardly detectable following PAGE of liver extracts of mice fed on the AIN 93 diet, which could account for the reduction in total SOD activity observed in Table 2. This is an important result, since the increased concentration of vitamin E and possibly other compounds of the AIN 93 diet clearly affected specific SOD isoforms, one located in the mitochondria (SOD II) and more strongly one located in the cytosol (SOD V), since the latter accounts for a higher SOD activity when compared to SOD II. Within the cell, vitamin E partitions into the hydrophobic core of the various cell membranes, including the inner and outer membrane of the mitochondria [25], although the relative concentration of vitamin E differs from one membrane to another [26]. Furthermore, *α*-tocopherol supplementation in human subjects and animal models has been shown to decrease lipid peroxidation and superoxide production by impairing the assembly of NADPH oxidase, as well as by decreasing the expression of scavenger receptors (SR-A and CD36) [27], to which our results appear to match such a possibility. The reduction observed in SOD activity in the livers of animals subjected to the increased vitamin E diet also suggests that the production of the superoxide radical is likely to be diminished more likely in the cytosol and the mitochondria, which agrees and can be clearly correlated to the specific depletion of SOD II and SOD V. Moreover, vitamin E has also been shown to prevent the induction of metallothionein synthesis as well as lipid peroxidation in the liver of mice administered the mitochondrial inhibitor 2,4-dinitrophenol [28], which agrees with the findings observed here of the depletion of the specific SOD isoforms and reduction in lipid peroxidation. Moreover, Fe-SOD isoforms, which can be found in living cells, but not necessarily in all living organisms, were not detected following PAGE in this work.

The enzymes CAT and GSH-Px are part of the next step of the antioxidant defense mechanism, converting H_2_O_2_ to water [29]. Alper et al. [30] reported a decrease in CAT activity in rats fed with a diet deficient in vitamin E. The authors suggested that the decrease in CAT activity might be due to the suppression of heme biosynthesis. Heme is a prosthetic group that consists of an iron atom contained in the center of a heterocyclic organic ring termed porphyrin, which is present in the molecule of CAT, and is synthesized in the liver and erythroid tissues [31]. Studies with animals deficient in vitamin E showed a decrease in hepatic activity of heme proteins such as CAT and microsomal cytochrome P450 and *b*_5_[30]. In this study, there was a significant increase in CAT activity in the livers of mice grown on the AIN 93 diet, which contained 2.5 fold higher more vitamin E than the commercial diet, supporting a role for this vitamin in improving the rate of removal of H_2_O_2_ in metabolically normal animals. Thus, the higher CAT activity observed in animals fed with the AIN 93 diet could explain the lower concentration of H_2_O_2_ observed (Figure 1B). Similar results were described by Ryan et al. [32] in a study on the muscles of rats fed with supplemented vitamin E or normal non-supplemented rat chow. GSH-Px can act directly on H_2_O_2_, however, this enzyme also acts in the inactivation of organic hydroperoxides [33]. There was little difference in the activity of GSH-Px in the livers of the mice fed with the two different diets. Similar results were reported in a study with rats fed with diets supplemented with vitamin E and a control diet [25]. GR is an enzyme that is used for the regeneration of reduced glutathione from oxidized glutathione, especially when the cell is exposed to free radicals. Shireen et al. [34] working with rats fed with an AIN 76 diet and AIN 76 supplemented with vitamin E and C, demonstrated that there was a significant increase in the activity of both GSH-Px and GR, in the animals fed with the vitamin supplemented diet. In this study, the activity of GR in the livers of animals fed with the AIN 93 diet was significantly lower than in animals fed with the commercial diet, suggesting that the AIN 93 diet group has a lower demand for reduced glutathione in the cellular defense mechanisms, or that GR may not have a major role as a defense enzyme under the conditions tested. A wide range of antioxidant enzymes exist in order to keep the redox state of the cell, so they are important not only under normal metabolic conditions, but also when dealing with stressful conditions. Although interesting responses in enzyme activities were detected in this research, the role of other peroxidases or antioxidant enzymes cannot be ruled out and should be considered in future research.

Before any major conclusions can be drawn, it is important to state that this study did not investigate varying concentrations of vitamin E or of any other individual components of the diets, which limits the impact of the results and how they are correlated with the major changes observed. Even with such a limitation, the data obtained are not invalidated. However, future studies should be performed in a way that varying concentrations of vitamin E and other components of both diets are tested so that the effects on the antioxidant responses are more clearly understood and the putative key role of vitamin E is confirmed. Based on the composition of both diets, it cannot be ruled out that other components may have had an indirect effect on mouse metabolism, which might have resulted in a response by the antioxidant system. Nevertheless, we must state that the fact that a high vitamin E concentration was used and that among the components of the diet vitamin E is clearly the only direct and the major non-enzymatic antioxidant included, we may assume that Vitamin E appears to have an important participation in the responses observed.

## Conclusion

In conclusion, our results suggest that the different composition of the two diets in particular the one with vitamin E included, with or without the co-participation of other diet components, appears to have a considerable effect on the defense mechanism against free radicals generated during normal metabolism in male Swiss strain mice. One key aspect of such an effect is likely to be the generation of the superoxide radical in the mitochondria and cytosol, based on reduction in activity of specific isoforms of SOD present in the liver of animals fed on the AIN 93 diet. Therefore, the results further suggest that a diet rich in vitamin E may naturally give extra protection to the liver against any oxidative stress condition that may occur, leading to excess ROS production, as a result of metabolic changes produced if the animal is subjected to a stressful condition.

## Abbreviations

AIN: American institute of nutrition; CAT: Catalase; GR: Glutathione reductase; GSH-Px: Glutathione peroxidase; MDA: Malondialdehyde; ROS: Reactive oxygen species; SOD: Superoxide dismutase.

## Competing interests

The authors declare that there is no conflict of interest in the dissemination of this data.

## Authors’ contributions

ACC participated in the acquisition of data, performed the statistical analysis, interpreted the data and drafted the manuscript. LFV participated in conducting the animal experiments and the acquisition of data. FRC participated in the acquisition of data, interpreted the data and revised the manuscript. SMA and RAA participated in the revision of the manuscript and RMNB conceived the study, determined the design, interpreted the data and drafted the manuscript. All authors read and gave final approval for the version submitted for publication.
